# Lower socioeconomic status is associated with premature brain aging

**DOI:** 10.1016/j.neurobiolaging.2023.06.012

**Published:** 2023-06-23

**Authors:** Natalie Busby, Sarah Newman-Norlund, Sara Sayers, Roger Newman-Norlund, Janina Wilmskoetter, Chris Rorden, Samaneh Nemati, Sarah Wilson, Nicholas Riccardi, Rebecca Roth, Lisa Johnson, Dirk B. den Ouden, Julius Fridriksson, Leonardo Bonilha

**Affiliations:** a Department of Communication Sciences and Disorders, University of South Carolina, Columbia, SC, USA; b Department of Psychology, University of South Carolina, Columbia, SC, USA; c Department of Neurology, Medical University of South Carolina, Charleston, SC, USA; d Department of Neurology, Emory University, Atlanta, GA, USA

**Keywords:** Brain age, Socioeconomic status, Brain Health, Health, Aging

## Abstract

**Background:**

Premature age-related brain changes may be influenced by physical health factors. Lower socioeconomic status (SES) is often associated with poorer physical health. In this study, we aimed to investigate the relationship between SES and premature brain aging.

**Methods:**

Brain age was estimated from T1-weighted images using BrainAgeR in 217 participants from the ABC@UofSC Repository. The difference between brain and chronological age (BrainGAP) was calculated. Multiple regression models were used to predict BrainGAP with age, SES, body mass index, diabetes, hypertension, sex, race, and education as predictors. SES was calculated from size-adjusted household income and the cost of living.

**Results:**

Fifty-five participants (25.35%) had greater brain age than chronological age (premature brain aging). Multiple regression models revealed that age, sex, and SES were significant predictors of BrainGAP with lower SES associated with greater BrainGAP (premature brain aging).

**Conclusions:**

This study demonstrates that lower SES is an independent contributor to premature brain aging. This may provide additional insight into the mechanisms associated with brain health, cognition, and resilience to neurological injury.

## 1. Background

Premature brain aging is typically denoted as age-related brain atrophy occurring out of proportion to chronological age (Cole et al., 2018). Individuals with premature brain age (i.e., with a higher brain age gap) have more cortical atrophy than would be expected based on their chronological age, suggesting an early compromise of brain structures (Cole et al., 2017). For this reason, brain aging can be more informative about the status of brain tissue compared with chronological age alone. Premature brain aging is becoming increasingly recognized as an important factor related to lower cognition among healthy older adults (Franke & Gaser, 2019).

To date, brain aging research has provided converging evidence indicating a strong relationship between premature brain age and cardiovascular risk factors and other negative health-related conditions. For example, Franke et al. noted that diabetes is associated with premature brain aging (Franke et al., 2013). Hedderich et al. observed that a higher brain age gap was also observed in adults who were born prematurely (Hedderich et al., 2021). Moreover, Etherton et al. found that higher body mass index (BMI) and presence of hypertension and diabetes were associated with poorer brain health (Etherton et al., 2016). Nonetheless, the impact of environmental variables on brain aging remains less well understood. More specifically, socioeconomic status (SES) is a measure of an individual’s economic access to resources and social position in relation to others (Franke & Gaser, 2019; Hedderich et al., 2021). Lower SES has been shown to be a reliable correlate of poor physical health (Matthews & Gallo, 2010; Wang & Geng, 2019), possibly due to a multifactorial interaction with access to health care or nutritional resources. Studies have investigated childhood SES and structural brain development (Brito & Noble, 2014). Lower SES is commonly associated with cardiovascular risk factors (Davari et al., 2019), but it is unclear if lower SES is an independent factor related to premature brain aging.

Therefore, in this study, our aim was to investigate the relationship between SES and brain aging, with the hypothesis that individuals with lower SES would be more likely to experience premature brain aging even when controlling for cardiovascular risk factors. Understanding this relationship could provide insight into the mechanisms accounting for premature brain age.

## 2. Methods

### 2.1. Participants

Participants (N = 217) were part of the Aging Brain Cohort at the University of South Carolina (ABC@UofSC) Repository (Newman-Norlund et al., 2021), an ongoing original cross-sectional cohort. ABC@UofSC focuses on healthy aging and therefore has the following exclusion criteria: anyone who has previously had a stroke, a diagnosis of a neurodegenerative disease, acute or chronic conditions that would limit their ability to participate, any severe current illnesses (e.g., cancer), any psychiatric diagnosis (e.g., schizophrenia), or anyone with a BMI of > 42 kg/m^2^. All participants who had complete demographic and behavioral data and had completed a brain scan were included in this study. Institutional review board approval was obtained, followed by written informed consent provided by all participants at enrollment.

### 2.2. Neuroimaging acquisition and preprocessing

All participants underwent the same magnetic resonance imaging scanning protocol on a Siemens Trio 3T scanner with a 20-channel head coil. T1-weighted images were used for brain age estimation and were acquired using the following parameters: T1-weighted imaging (MP-RAGE) sequence with 1 mm isotropic voxels, 256×256 matrix size, 9° flip angle, and 92-slice sequence with repetition time = 2250 ms, inversion time = 925 ms, and echo time = 4.11 ms.

### 2.3. Brain age estimation

Brain age estimation was performed based on T1-weighted images using the BrainAgeR analysis pipeline (github.com/james-cole/brainageR) (Cole et al., 2018). In the BrainAgeR pipeline, T1-weighted images were segmented and normalized using SPM12’s DARTEL toolbox (Ashburner & Friston, 2005). Then, probabilistic tissue maps were visually inspected by a neurologist (LB) to ensure the quality of the segmentation. Gray and white matter probabilistic tissues were entered into a machine learning algorithm using a pretrained Gaussian regression model implemented in R-package Kernlab to estimate brain age.

**Fig. 1.**
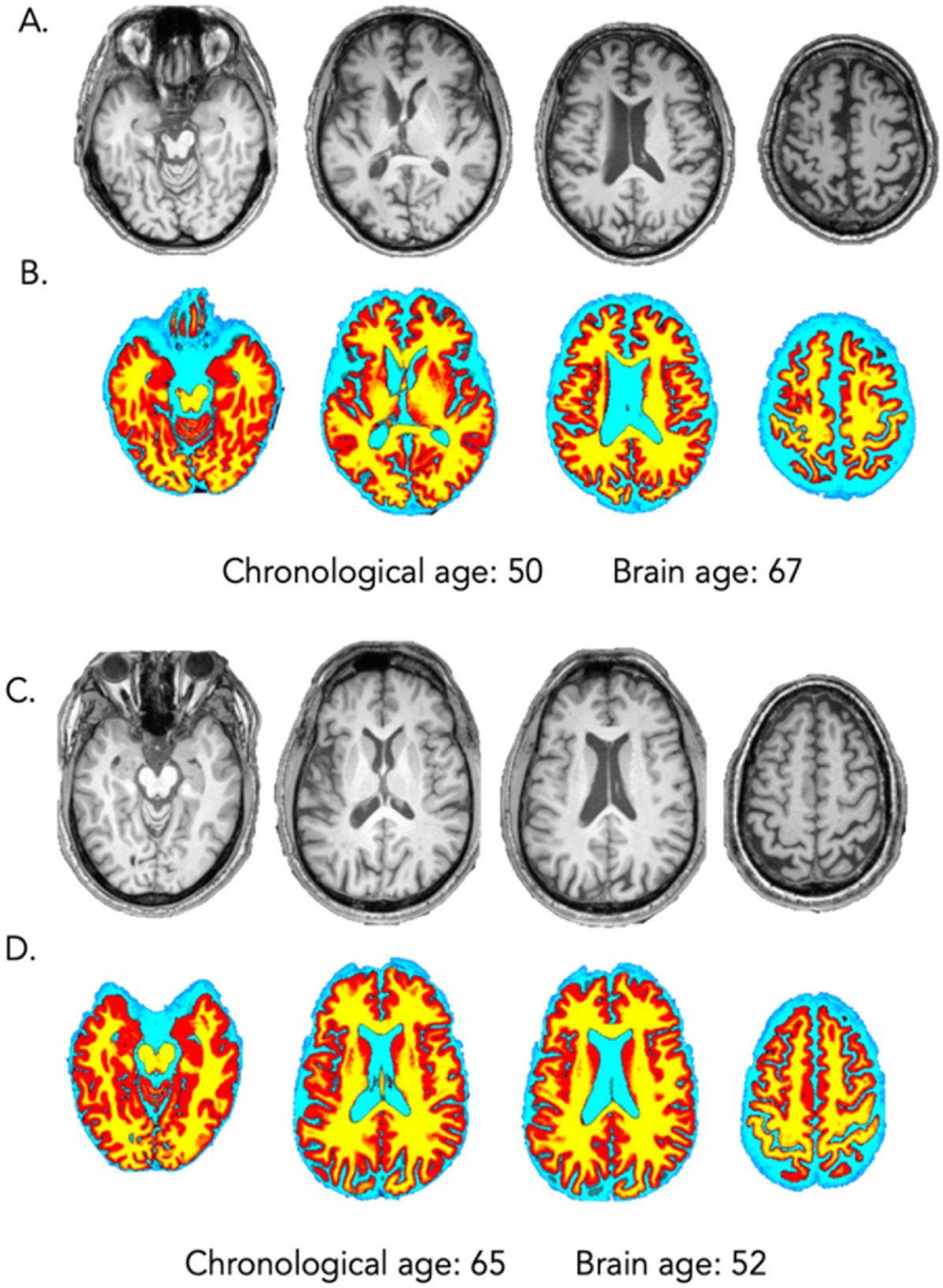
A and B show premature brain aging, where the participant has a chronological age of 50, but an estimated brain age of 67, (A) T1-weighted image and (B) segmented T1 showing gray matter (red), white matter (yellow), and cerebrospinal fluid (blue). C and D show a younger brain age than chronological age, where the participant has a chronological age of 65 and a brain age of 52, (C) T1-weighted image and (D) segmented T1 showing gray matter (red), white matter (yellow) and cerebrospinal fluid (blue).

The relative difference between estimated brain age and chronological age (brain age gap: BrainGAP) was determined by subtracting an individual’s chronological age from their estimated brain age. Therefore, the BrainGAP is the measure of premature or delayed brain age beyond chronological age. Positive values suggest that the estimated brain age was older than the actual chronological age of the participant (i.e., premature brain aging; Fig. 1).

### 2.4. Socioeconomic status

Total household income and number of individuals in the household along with the cost of living for Columbia, SC metropolitan area were used to determine class tier as a measure of SES. We calculated the income tier using Pew Research Center’s income calculator using data from the 2018 US Census. Possible scores ranged from 1 (low) to 3 (high).

### 2.5. Cardiovascular risk factors

At the time of magnetic resonance imaging scanning, participants were given a detailed questionnaire addressing medical history concerning variables known to be related to brain health (e.g., yes/no questions about diabetes and hypertension, and BMI was calculated [based on height and weight]). Questionnaire responses were corroborated by medical records, where available.

### 2.6. Statistical Analysis

To investigate the relationship between chronological age, brain age, and BrainGAP, correlations were conducted between the three factors. A multiple regression model was then used to investigate the relationship with SES. It is likely that SES is associated with cardiovascular risk factors (e.g., BMI, diabetes, and hypertension), and brain age has been previously associated with demographic variables, such as sex, race, and education, so these variables were also included in the analysis. Therefore, the dependent variable was BrainGAP, and the independent variables were SES, chronological age, BMI, diabetes (yes/no), hypertension (yes/no), sex, race, and years of education. To account for age separately, an additional linear regression was run where the dependent variable was BrainGAP and the independent variable was age. The residuals of this model were saved and were then used as the dependent variable in a subsequent regression model with BMI, diabetes, hypertension, sex, race, and years of education as independent variables. Statistical analyses were conducted using RStudio v1.4.1106, and graphs were created using the GGPLOT2 package.

## 3. Results

**Fig. 2.**
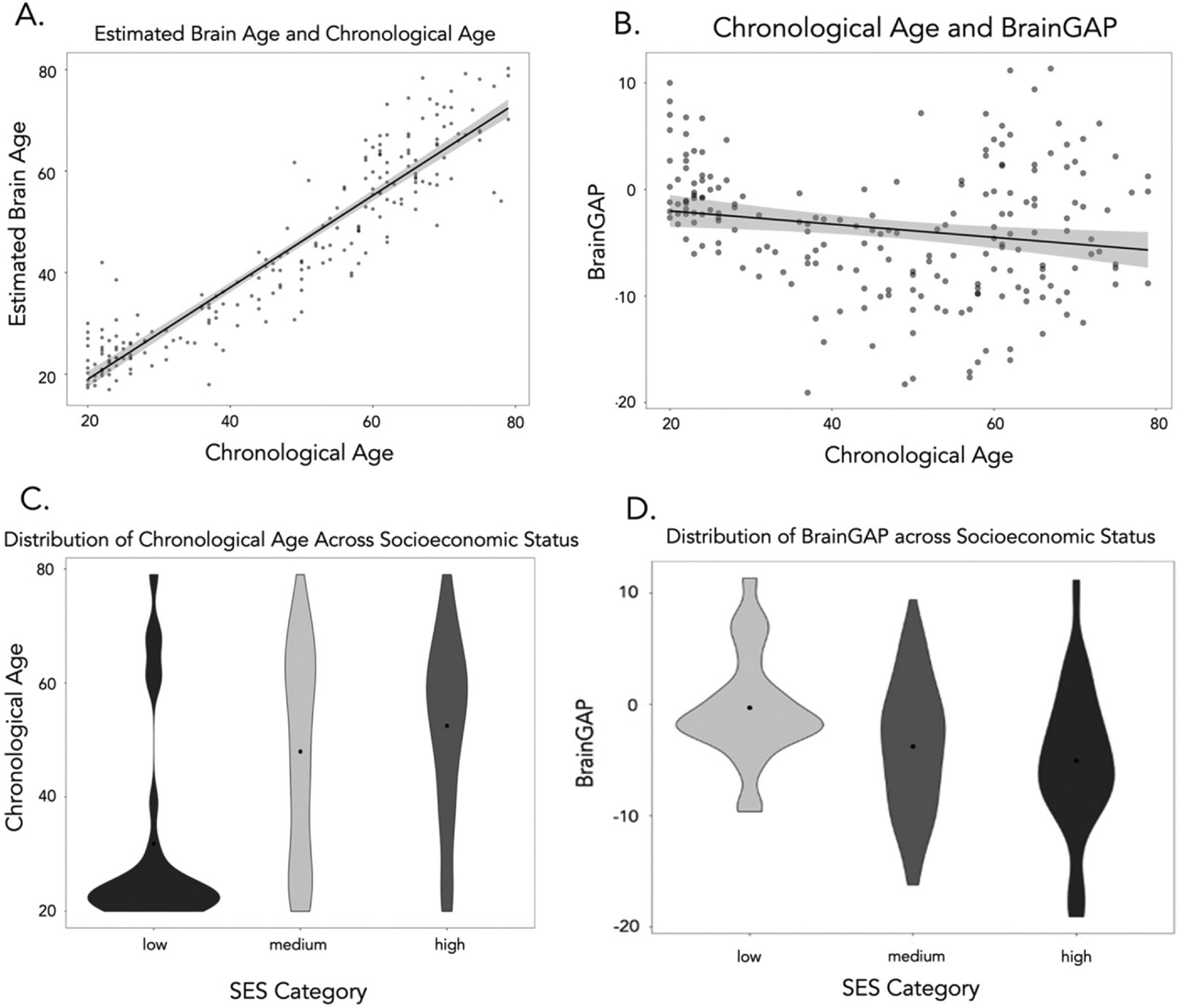
(A) Scatterplot showing the relationship between estimated brain age and chronological age. (B) Scatterplot showing the relationship between BrainGAP and chronological age. Positive BrainGAP scores indicate a higher brain age than chronological age (i.e., premature brain aging). (C) Violin plot to display the distribution of chronological age across SES categories. (D) Violin plot to display the distribution of BrainGAP scores across SES categories. Positive BrainGAP scores indicate a higher brain age than chronological age (i.e., premature brain aging). SES, socioeconomic status.

Participants had chronological ages between 20 and 79 years (mean = 47.44, standard deviation [SD] = 18.33) and estimated brain ages between 16.95 and 80.22 years (mean = 43.72, SD = 17.80). There was a significant positive correlation between chronological age and predicted brain age (Pearson: *r*(216) = 0.936, *p* < 0.001; see Fig. 2).

The average BrainGAP was −3.72 (SD = 5.96, range = −19.07 to +11.34). Fifty-five participants (24.35%) had greater brain age compared to chronological age (i.e., premature brain aging). There was a significant negative correlation between chronological age and BrainGAP (Pearson: *r*(216) = −0.245, *p* < 0.001; Fig. 2). It is important to note that there was a significant positive correlation between age and SES in this study (*r*(216) = 0.295, *p* < 0.001), where individuals with lower SES were typically younger. See Appendix 1 for a full breakdown of health and demographic information for each SES group.

The multiple regression model was significant (*R^2^* = 0.13, *F* (8207) = 4.02, *p* < 0.001), and three variables were statistically significant: chronological age (*p* = 0.002), sex at birth (*p* = 0.005), and SES (*p* = 0.046; see Table 1 for full results). These results reveal that those with greater BrainGAP (i.e., older brain age compared to chronological age) were, on average, younger, male, and had a lower SES. See Fig. 2D for a distribution of BrainGAP across SES categories. Tests to see if the data met the assumption of collinearity indicated that multicollinearity was not a concern (age: tolerance = 0.739, variance inflation factor [VIF] = 1.352; sex: tolerance = 0.988, VIF = 1.013; SES: tolerance = 0.859, VIF = 1.165; BMI: tolerance = 0.913, VIF = 1.096; hypertension: tolerance = 0.783, VIF = 1.277; diabetes: tolerance = 0.890, VIF = 1.124; education: tolerance = 0.941, VIF = 1.063; race: tolerance = 0.914, VIF = 1.094).

## 4. Discussion

Brain age is a measure of aging-like brain tissue atrophy that can be more informative regarding cognitive function and resilience to neurological injury compared with chronological age (Anatürk et al., 2021). Given the known relationship between SES and health factors such as the increased likelihood of cardiovascular diseases, we investigated the association between SES and accelerated brain aging. Our results show low SES is independently associated with brain aging, even when accounting for chronological age and cardiovascular risk factors. The reason for this relationship is likely multifactorial. Nonetheless, our data suggest that lower SES is associated with premature decay in brain structures.

While studies have previously investigated the role of SES on brain structure, almost all the focus has been on the relationship between childhood SES and brain development (e.g., Staff et al., 2012; Hackman & Farah, 2009, etc.). It is indeed possible that individuals with smaller or less developed brains in childhood are more likely to have a smaller brain into adulthood and therefore would appear to have premature brain aging. However, although childhood and adolescence are important times of brain development with long-lasting implications, it is also important to acknowledge that structural brain changes occur with older age as a result of the aging process, and there could be an interaction between environmental factors and brain aging. In our study, we found that approximately a quarter of the studied individuals exhibited premature brain aging.

Our results demonstrated the impact of SES on physical brain health and the importance of considering SES as a factor with neurobiological relevance during mechanistic or interventional studies. This may be of particular importance in relationship with studies investigating the determinants of cognitive factors or resilience to injury such as in the case of strokes or other pathologies. Aging is one of the most important determinants of brain plasticity and the relationship between brain aging (as a tissue-related factor beyond chronological age) appears to be as important, if not more so, than chronological age for neurological health. Our results confirmed our hypothesis regarding SES and premature brain aging, and these results could inform future research in the field.

## 5. Limitations

A limitation of this study is that there is an uneven distribution of age across the SES categories, with a higher proportion of low SES participants in the 20–25 years age range. Given that SES was a significant predictor of BrainGAP with age in the model, this suggests that SES still influences physical brain health irrespective of age. However, future studies should ensure an equal distribution of age across SES categories to confirm this. Another limitation is the use of self-reported measures of health used within this study. ABC@UofSC are collecting comprehensive laboratory panels and health information from participants; however, during the COVID-19 pandemic—although ABC@UofSC was able to continue with recruitment through online or socially distanced testing—close-contact data (i.e., taking blood for laboratory panels or measuring blood pressure) were not possible. Therefore, full health panels were not available for 52% of participants included in this study. Future studies should utilize detailed health information to support these results, suggesting factors such as diabetes or hypertension were not significantly associated with BrainGAP. Additionally, we did not include any motion or quality metrics, although all native space T1-weighted scans and segmented tissue maps were visually inspected (by LB) and were run through the CAT12 automated quality assurance pipeline to ensure high data quality and accurate segmentation, and CAT12 unified scan numbers. Finally, although many studies of premature brain aging focus on age-related atrophy out of proportion to chronological age, it is also important to note that inter-individual differences (e.g., genetics) or childhood factors (e.g., differences in brain development) may also influence brain structure later in life. Therefore, future studies should investigate these alternative explanations for greater brain age alongside atrophy-focused hypotheses.

## Figures and Tables

**Table 1:** Results from the multiple regression model

Factor	Estimate	Standard error	*t* value	*p* value	Percentage of variance explained
Age	−0.085	0.026	−3.209	0.002*	4.57
Sex at birth (male: 1, female: 2)	−2.726	0.961	−2.834	0.005*	3.60
SES	−1.391	0.701	−1.982	0.048*	1.79
BMI	−0.159	0.084	−1.898	0.059	1.65
Hypertension (presence)	2.011	1.12	1.795	0.074	1.48
Diabetes (presence)	1.008	1.744	0.578	0.564	0.16
Years of education	0.274	0.493	0.556	0.579	0.14
Race	0.137	0.582	0.236	0.814	0.14

The results of the second linear regression model reflected the first, where sex (*p = 0.005) and SES (p = 0.037) were the only two significant predictors. A full breakdown of these results can be found in* Appendix 2.

* *p* < 0.05.

Key: BMI, body mass index; SES, socioeconomic status.

## Data Availability

The data that support the findings of this study are available from the corresponding author upon reasonable request.

## Appendix A

See Tables A1 and A2.

**Table A1:** Health and demographic information for all participants and for each SES group

Factor	All participants	Low SES	Medium SES	High SES
Age	47.44	35.53	47.14	53.49
Brain age	43.72	33.98	43.73	48.19
BrainGAP	−3.72	−1.55	−3.41	−5.31
BMI	27.53	26.90	28.06	26.83
Education	3.30	2.97	3.28	3.48
Sex at birth (% male)	23.04	26.67	21.49	24.24
Type 2 diabetes (% presence)	6.48	3.33	5.00	10.61
Hypertension (% presence)	23.14	16.67	23.97	24.24
High cholesterol (% presence)	26.85	16.67	26.45	32.31
History of heart palpitations/irregular heartbeat	9.26	10	9.92	7.70
Hyperlipidemia	2.32	0	3.31	1.54

Key: BMI, body mass index; SES, socioeconomic status.

**Table A2:** A secondary linear regression model where the dependent variable is the residuals of a regression between BrainGAP and age (dependent variable: BrainGAP, independent variable: age)

Factor	Estimate	Standard error	*t* value	*p* value
Sex	−0.432	0.152	−0.190	0.005*
SES	−0.221	0.106	−2.095	0.037*
BMI	−0.025	0.013	−1.923	0.056
Hypertension (presence)	0.318	0.172	1.848	0.066
Diabetes (presence)	0.159	0.273	0.538	0.561
Years of education	0.043	0.078	0.557	0.578
Race	0.022	0.090	0.245	0.807

Key: BMI, body mass index; SES, socioeconomic status.

* p < 0.05.

## Abbreviations:

ABC@UofSC: AgingBrain Cohort at the University of South Carolina
BMI: body mass index
SD: standard deviation
SES: socioeconomic status
VIF: variance inflation factor
